# On the Influence of Action Preference on Female Players' Gaze Behavior During Defense of Volleyball Attacks

**DOI:** 10.3389/fspor.2020.00006

**Published:** 2020-02-04

**Authors:** Tim Lüders, Jörg Schorer, Florian Loffing

**Affiliations:** Institute of Sport Science, Carl von Ossietzky University of Oldenburg, Oldenburg, Germany

**Keywords:** anticipation, contextual information, eye-tracking, decision behavior, congruence, decision making, situational probability

## Abstract

Knowledge of an opponent's action preference may affect visual anticipation of their action outcome. Specifically, if an opponent acts according to their purported preference, anticipation may be facilitated. Conversely, if an opponent does not act according to their purported preference, anticipation may be unaffected or even harmed. The underlying perceptual-cognitive mechanisms of that effect, however, remain unclear. Here we tested the hypothesis that players might change their gaze behavior once provided with preference information. To this end, 27 female volleyball players anticipated the direction of attacks in two test blocks with 40 videos each. Videos were shown on a large screen and stopped 240 ms prior to hand-ball-contact. Participants simulated defensive reaction while their gaze was recorded using a mobile eye-tracker. One female attacker directed 75% of shots diagonally (25% longline), while another female attacker distributed shots equally to both directions. After block one, half of the participants were informed that either both attackers preferred diagonal shots in 75% of occasions (group preferred) or that both attackers distributed shots equally across directions (group non-preferred). Analysis of decision behavior (i.e., proportion of diagonal decisions), but not prediction accuracy (i.e., proportion of correct predictions), revealed that those instructions led both groups decide differently according to the purported preferences from block 1 to block 2. Analysis of gaze behavior did not reveal group-specific effects across blocks or attackers with/-out action preference. Findings underline the influence of contextual information on anticipation, but they leave open whether the availability of contextual information similarly affects gaze behavior.

## Introduction

Anticipation in team sports such as volleyball is considered a crucial component for success (Allard and Starkes, 1980; Abernethy, 1987). At the latest since the penalty shootout at the 2006 World Cup between Germany and Argentina, when German goalkeeper Jens Lehmann was given leaflets about the possible shooting directions of the Argentinian shooters, public awareness of the potential utility of contextual information in sports has increased and its added value for anticipation scientifically questioned (Cañal-Bruland and Mann, 2015). Here, contextual information is considered as supplementary information from which probabilities for action arise apart from an actor's kinematics. By now, growing evidence suggests that, in addition to kinematic cues provided by an opponent's movement, contextual information also influences anticipation performance (Loffing and Cañal-Bruland, 2017). For example, game score (Farrow and Reid, 2012), individual action preferences (Navia et al., 2013; Mann et al., 2014; Helm et al., 2020), pattern of previous outcomes (Loffing et al., 2015b), or an opponent's on-court position (Loffing and Hagemann, 2014; Loffing et al., 2016; Huesmann and Loffing, 2019) were found to affect anticipation of an opponent's action intention in different sports. The investigation of the influence of an opponent's action preference on anticipation is particularly interesting here, because in high-performance sports data about opponents is collected and processed in the run-up to a game for an in-depth match planning (McGarry et al., 2013). Data is also even generated during a match, made available to coaches and further passed on to the athletes in timeouts (Zetou et al., 2008). The idea behind providing athletes with opponent-related information during competition is to facilitate their performance.

Mann et al. (2014) studied the effect of an opponent's action preference on skilled female handball goalkeepers' visual anticipation of throw direction in 7 m handball penalties before and after a short-term training intervention. In the training intervention, one group (PR) was shown videos of two shooters who had an action preference for one corner, the other group (N-PR) was shown videos of the same shooters who distributed the balls equally across corners. Thus, contextual information was varied through action preference. In the tests, the same two shooters were shown, with one of them preferentially throwing to the same corner as in the training intervention and the other having no preference. Pre- to post-test comparison revealed that contextual information conveyed during training affected directional anticipation. Participants in the PR-group decided more often on the (assumed) preferred corner in the post- than the pre-test. Moreover, from pre- to post-test accuracy increased against the thrower with congruent behavior (i.e., preference in tests and training), but it decreased against the thrower with incongruent behavior (i.e., no preference in tests but in training). Performance of the N-PR-group did not markedly differ from pre- to post-test, suggesting that preference information picked-up during training is likely to explain performance changes in the PR-group (for similar findings see e.g., Loffing et al., 2015b; Gredin et al., 2018; Runswick et al., 2019).

The perceptual-cognitive mechanisms underlying the above-mentioned effect of action preference on anticipation have not yet been satisfactorily clarified. One explanatory approach suggests a confirmation bias whereby information that meets expectations is specifically selected and processed, while information that does not meet expectations is ignored (Nickerson, 1998). Because contextual information is available before anticipation-relevant kinematics unfold (Cañal-Bruland and Mann, 2015), this could trigger an expectation that leads to a confirmation bias for incongruent events, ultimately resulting in a performance decrease. In tentative support of this idea, using an anticipation task in cricket Runswick et al. (2019) found that skilled batters were more susceptible to the effect than less-skilled batters, possibly because the former are more reliant on and better in using contextual information.

The manipulation of contextual information might also result in a variation of visual-perceptual measures. McRobert et al. (2011) showed in a cricket-batting task that skilled participants under high context conditions (i.e., same bowler in six consecutive trials) had shorter mean fixation duration than under low context conditions (i.e., different bowlers in consecutive trials). According to the authors' apostrophe *post-hoc* explanation experts know where to find cues for the expected action and can then pick them up more efficiently, which is reflected in the shorter fixation duration. No differences in the mean number of fixations were observed. This study, however, did not compare gaze behavior in congruent vs. incongruent situations. Gredin et al. (2018) did so by asking expert and novice soccer players to anticipate an attacker's running or pass direction in a defensive situation in football. In addition to the player in possession of the ball, another opponent was shown who was covered by another teammate. In one test condition comprising six sub-blocks, information about the ball dribbling player's action tendency for either passing to the other player or continued dribbling was communicated explicitly both orally and visually on a screen before each sub-block. In another test condition, stimuli were identical to the other condition but no explicit information on action tendencies were given prior to sub-blocks. Examination of participants' visual dwell time revealed that experts, but not novices, looked less at the player in possession of the ball but more at the two players (i.e., opponent and teammate) without the ball when provided with explicit contextual information relative to when not. This was particularly evident in the first, but not the second half of a trial.

Taken together, both studies provide preliminary evidence that gaze behavior may vary depending on context. However, it remains unclear whether gaze behavior differs between congruent and incongruent events and whether such possible effect is reflected in anticipation performance differences as well. The aim of this study was to investigate whether the provision of information about an opponent's action preference has the purported positive effect on anticipation and to explore the effect of action preference on gaze behavior using the example of defense in volleyball attacks under congruent or incongruent conditions. The focus of our gaze analysis was on the number of fixations and the duration of the last fixation. The number of fixations was considered relevant as the pick-up of visual information occurs during fixations. The duration of the last fixation was chosen because the most important kinematic information of a movement is available in the final phase of a movement (Alder et al., 2014; Loffing et al., 2015a). Using a similar experimental design as Mann et al. (2014), here skilled volleyball players were asked to simulate the defense of attacks against opponents with and without action preference in two consecutive blocks. Between blocks, one group (PR) received information that both attackers had an action preference in favor of the diagonal direction, while another group (N-PR) received information that the attacks were evenly distributed. For group PR but not group N-PR, we expected decision behavior to change in favor of contextual information and prediction accuracy to increase for congruent events and to decrease for incongruent events (Mann et al., 2014; Loffing et al., 2015b; Gredin et al., 2018; Runswick et al., 2019). Regarding gaze measures, due to the exploratory nature of this study with regard to possible effects at the visual-perceptual level, no hypotheses were formulated a priori.

## Method

### Participants

A total of 27 female volleyball players (*M*_*age*_ = 24.8 years; *SD* = 4.7) from the second to fifth German volleyball league took part in the study. At the time of testing, they had an average of 12.9 years of playing experience (*SD* = 4.7) and completed, on average, 3 training units per week (*SD* = 0.7). The final sample was restricted to 24 participants because three participants had to be excluded prior to data analysis^1^. The study was carried out in accordance with the recommendations of and the protocol was approved by the local Commission for Research Impact Assessment and Ethics at the Carl von Ossietzky University of Oldenburg. All subjects gave written informed consent in accordance with the Declaration of Helsinki.

### Test Stimuli

**Figure 1:**
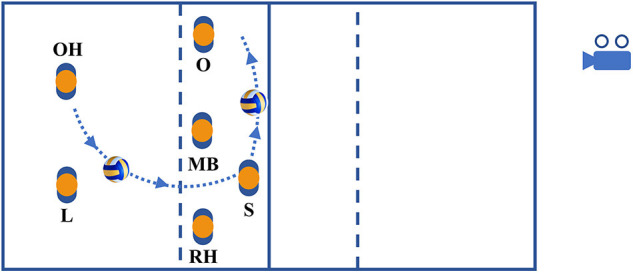
Representation of the play situation on the field with ball path (dotted line) and player positions (S, setter; O, opposite; OH, outside hitter; L, libero; RH, right side hitter; MB, middle blocker).

The test stimuli were videos of volleyball attacks performed by two different female players from the fourth German volleyball league. The attacks were embedded in a real game situation. The ball was taken by the outside hitter and played to the setter. The setter set the ball to the opposite (attacker) who hit the ball either longline or diagonal. Attackers were supposed to attack as if in a real game situation. Attack situations were recorded using a video camera (Panasonic HC-V270), which was positioned on a tripod (1.5 m high) two meters behind the baseline and one meter away from the sideline in the other half of the field (for an illustration see Figure 1).

A total of 25 video sequences were prepared for each attacker, 10 longline strokes and 15 diagonal strokes each. The video sequences started at the moment of ball-hand-contact during ball reception of the outside hitter and were occluded 240 ms before ball-hand-contact of the attacking player. The occlusion point was determined based on pilot testing with three skilled volleyball players (all different to those tested in the main experiment), who anticipated the ball flight direction for attacks occluded at different time points. In the main study, temporal occlusion at 240 ms before hand-ball-contact was chosen as accuracy of anticipation under that condition was found sufficiently above chance but below perfect performance in the pilot study. So, that temporal occlusion condition was expected to not lead to floor or ceiling effects in accuracy in the main study, thus leaving enough space for accuracy variation between test blocks as a consequence of the manipulation of the opponents' action preference.

### Apparatus

During testing, videos were back-projected (Acer X137WH) onto a rear projection screen (Stumpfl Flex Rear MO, 3 × 4 m) with a size of 2.17 × 3.85 m. Participants stood two meters in front of the screen so as to create a game situation as real as possible in terms of viewing angle. Participants' gaze behavior was recorded using the SMI Eye Tracking Glasses 2.0. Glasses were calibrated using a three-point calibration. To this end, participants stood two meters in front of a wall with four markers attached to it. The markers were arranged in a square with a side length of one meter, with the middle of the square being 1.7 m above ground. Participants were instructed to keep their head stable and only move their eyes toward each marker. The calibration was performed before the practice trials prior to the first block and checked before the second block. During testing, the eye-tracker's calibration was continuously controlled by asking participants to fixate a small cross on the screen prior to each trial.

### Procedure

In the experiment the participants went through three phases: test block 1, an information phase and test block 2 (see Table 1 for an illustration). For a given participant, blocks 1 and 2 were identical. In the tests, 20 videos of each attacker were presented in random order. The videos of the two different attackers were selected in such a way that one attacker had an equal distribution of shot directions (no action preference), while the other attacker hit 75% of the balls (i.e., 15 videos) in the diagonal direction (action preference). An attacker's action preference was counterbalanced across participants. In each group one attacker was shown whose attack direction matched with the information given between the blocks (congruent condition), while for the other attacker attack distribution did not match with the information given (incongruent condition).

**Table 1:** Experimental design.

**Group**	**Block 1**	**Information**	**Block 2**
N-PR	Attacker 1 with a distribution of 50%:50% (diagonal:longline)	Both attackers distribute strokes equally longline and diagonal (no preference)	Attacker 1 with a distribution of 50%:50%
	Attacker 2 with a distribution of 75%:25%		Attacker 2 with a distribution of 75%:25%
PR	Attacker 1 with a distribution of 50%:50%	Both attackers have a preference for diagonal strokes (in 75% of occasions)	Attacker 1 with a distribution of 50%:50%
	Attacker 2 with a distribution of 75%:25%		Attacker 2 with a distribution of 75%:25%

*Note that shot distribution in the tests was varied between the two attackers and counterbalanced across participants*.

Participants stood 2 m in front of the screen in a typical defensive position, wearing their volleyball specific clothes, and they were asked to put themselves into the game situation. The video sequences started once participants indicated they were ready for the next trial. Their task was to anticipate the direction of the attack by performing a defensive movement as in a real game and verbalizing the predicted stroke direction afterwards. The defensive movement should be timed as in a real game situation. The first direction of the defensive response was recorded by the experimenter. Participants were not given any performance feedback on any trial. Before the start of block 1, the participants underwent eight practice trials. Performance on those trials was not considered in later analysis. As for test trials no feedback was given to participants.

Between the two test blocks explicit information about the attackers' strike preference was given as text and graphically on the screen. Almost half of the participants were informed that both attackers had no action preference and hit as often diagonally as longline (group N-PR; *n* = 13). The other half of participants was informed that both attackers had an action preference in favor of diagonal strokes and that 75% of the attacks were struck diagonally (group PR; *n* = 11). Participants were allocated randomly to one of the two groups.

Block 2 was identical to block 1 in that the same videos were presented, however in a newly randomized order, and that the participants' task also was the same. Upon completion of block 2, participants completed a questionnaire in which they were asked, among others, to indicate what they thought was the particular aim of this study.

### Data Analysis

#### Anticipation Performance

Anticipation performance was operationalized by prediction accuracy and decision behavior. Prediction accuracy was measured as the proportion of correct direction prediction, ranging between 0 (no correct prediction at all) and 1 (perfect performance). Decision behavior, in turn, was determined as the proportion of decisions for diagonal strokes, again ranging from 0 (no diagonal prediction) to 1 (only diagonal predictions). Both variables were analyzed because changes in prediction accuracy must not necessarily go in the similar direction as changes in decision behavior and vice versa (Loffing and Hagemann, 2014; Mann et al., 2014). Both variables were subjected separately to a 2 (Group: PR vs. N-PR) x 2 (Block: block 1 vs. block 2) x 2 (Action Preference: attacker with vs. without action preference) mixed ANOVA with repeated measures on the last two factors. Alpha level was set to 0.05 for all tests.

#### Gaze Behavior

Gaze behavior was measured via the mean number of fixations across the full length of a video and the mean duration of the last fixation in a video. It was analyzed by the program “Begaze 3.7” from SMI. Both variables were subjected separately to a 2 (Group: PR vs. N-PR) x 2 (Block: block 1 vs. block 2) x 2 (Action Preference: attacker with vs. without action preference) mixed ANOVA with repeated measures on the last two factors. Alpha level was set to 0.05 for all tests.

## Results

Results from the post-experiment questionnaire revealed that five out of 24 participants revealed the study objective almost correctly, suspecting that the study aimed at investigating the influence of additional information on anticipation or gaze behavior. Two of these participants belonged to the N-PR group and three to the PR group.

### Anticipation Performance

**Figure 2:**
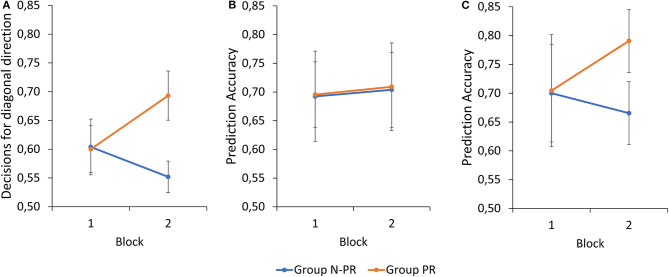
**(A)** Mean proportion of decisions for diagonal strokes as a function of Block and Group. Mean prediction accuracy against an attacker **(B)** without and **(C)** with an action preference as a function of Block and Group. Error bars denote 95% confidence intervals of the mean.

The 2 (Group) x 2 (Block) x 2 (Action Preference) ANOVA on *decision behavior* revealed a significant main effect for Group, *F*_(1, 22)_ = 6.22, *p* = 0.021, $\eta_{\text{p}}^{2}\;$ = 0.22. Overall, the group PR made more decisions in favor of the diagonal direction than the group N-PR. In addition, there was a significant Block x Group interaction, *F*_(1, 22)_ = 28.84, *p* < 0.001, $\eta_{\text{p}}^{2}\;=$ 0.57. In block 1, both groups decided equally often on diagonal strokes. In block 2, however, group PR decided considerably more often on diagonal shots (according to the information given in-between), whereas group N-PR made fewer diagonal decisions than in block 1 and compared to group PR (see Figure 2A). None of the remaining main effects or interactions were found to be significant.

For *prediction accuracy*, the descriptive data related to the attacker without an action preference do not show noticeable differences between blocks 1 and 2 irrespective of group (block 1: *M* = 0.694, *SD* = 0.128; block 2: *M* = 0.706, *SD* = 0.127; see Figure 2B). With regard to the attacker who had an action preference, descriptively the groups showed different developments from block 1 to block 2. Specifically, accuracy in group PR increased (block 1: *M* = 0.705, *SD* = 0.172; block 2: *M* = 0.791, *SD* = 0.097) and it decreased in group N-PR (block 1: *M* = 0.700, *SD* = 0.162; block 2: *M* = 0.665, *SD* = 0.105) (see Figure 2C). A 2 x 2 x 2 mixed ANOVA, however, did not reveal significant results for any main effect or interaction.

Exclusion of the five participants who suspected the study objective correctly from the above analyses does not lead to noticeable changes in the results for anticipation performance.

### Gaze Behavior

The 2 (Group) x 2 (Block) x 2 (Action Preference) ANOVA on the *number of mean fixations* revealed a significant main effect for Block, *F*_(1, 22)_ = 10.82, *p* = 0.003, $\eta_{\text{p}}^{2}\;$ = 0.33, and Action Preference, *F*_(1, 22)_ = 5.43, *p* = 0.029, $\eta_{\text{p}}^{2}\;$ = 0.20. Participants made more fixations in block 1 compared to block 2 (block 1: *M* = 7.45, *SD* = 1.38; block 2: *M* = 6.96, *SD* = 1.47) and they fixated more against the attacker with an action preference (*M* = 7.30, *SD* = 1.44) than when confronted with the attacker who had no preference (*M* = 7.11, *SD* = 1.45). None of the remaining main effects or interactions were found to be significant.

The 2 x 2 x 2 ANOVA on the *duration of the last fixation* revealed a significant main effect for Action Preference, *F*_(1, 22)_ = 5.93, *p* = 0.023, $\eta_{\text{p}}^{2}\;$ = 0.21. On average, participants made a longer last fixation against the attacker without an action preference (*M* = 910.95 ms, *SD* = 415.33 ms) as opposed to an attacker with an action preference (*M* = 831.08 ms, *SD* = 347.30 ms). None of the remaining main effects or interactions were found to be significant.

If the five participants who suspected the study objective correctly were excluded from the above analyses of gaze measures, the results would marginally change in that the main effect for Action Preference on *the number of mean fixations* (*p* = 0.102) and *duration of the last fixation* (*p* = 0.132) would no longer be significant.

A detailed list of all statistical values obtained from the analysis of all 24 participants is included in the Supplementary Materials.

## Discussion

Previous research suggests that contextual information can have a positive or negative influence on anticipation, depending on whether the occurring event is congruent or incongruent with the expected event based on prior contextual information (Mann et al., 2014; Loffing et al., 2015b; Gredin et al., 2018; Runswick et al., 2019). The underlying perceptual-cognitive mechanisms of this effect, however, remain vastly unclear. The aim of this study was to explore whether the provision of information on an attacker's action preference in volleyball would affect gaze behavior, apart from the hypothesized influence on anticipation performance, in a simulated volleyball defense situation.

With regard to anticipatory performance, it could be shown that the provision of information on action preference influenced decision behavior, as there was a block x group interaction. In the first block, both groups opted equally frequently for diagonal strokes, in the second block participants of group PR showed more frequent decisions in favor of the instructed direction. These findings go in line with other studies (Mann et al., 2014; Loffing et al., 2015b; Runswick et al., 2019) and support the idea that instructing about opponents' action preferences influences visual anticipation. While the exact mechanism underlying that effect remain unclear, recent explanations center around a Bayesian approach (Loffing and Hagemann, 2014; Gredin et al., 2019; Helm et al., 2020) or heuristics such as confirmation bias (Rajsic et al., 2015; Runswick et al., 2019).

However, such an effect was only found for decision behavior, but could not be confirmed at the level of statistical significance for prediction accuracy. Descriptively, information about an action preference facilitated prediction accuracy if the attacker actually had an action preference (congruent condition), but there was no detrimental effect on accuracy in case an attacker actually did not have a preference. These findings do not go in line with the findings of Mann et al. (2014), who found that knowledge about opponents' action preferences also influenced prediction accuracy. Overall, data on decision behavior suggest that action preference information is integrated in to predictions, because participants of group PR decided more often in favor of the instructed direction in block two; however doing so does not necessarily lead to considerably better (congruent condition) or worse (incongruent condition) decisions in terms of accuracy.

Gaze behavior measures were not found to statistically vary depending on Group in combination with other factors. Instead, only conceptually less relevant main effects for the factor Block on the number of fixations and for the factor Action Preference on both gaze measures were found. The Block effect possibly reflects participants' adaptation to the experimental task in general. Action preference information was given between test blocks and information differed between groups PR and N-PR. With this in mind and considering further that no feedback was provided to participants on the outcome of an attack during testing, the main effect for Action Preference and the absence of an interaction effect with Group and/or Block is difficult to explain. To avoid unreasonable speculation about possible explanations, we refrain from discussing these findings any further.

Two methodological issues that limit the interpretation of gaze measures need to be highlighted. First, attacks were always performed from the same field position (see Figure 1). This reduced uncertainty in the setter's action (i.e., where she set the ball to) and attack location, both possibly leading to low variability in participants' gaze behavior and ultimately lowering the chances of detecting potential variation in gaze measures between blocks and groups against attackers with and without an action preference. Second, the size of videos and the distance of participants to the projection screen were chosen such that the visual angle of the players in the videos was as close as possible to the visual angle of players viewed from a backcourt position in a real match. This resulted in a large part of an attacker being within the foveal and near peripheral field of view, which does not permit a meaningful detailed analysis of gaze orientation toward different parts of an attacker's bodily regions (Piras et al., 2010, 2014; Afonso et al., 2012; Schorer et al., 2013). These issues may need to be considered in future research on the visual-perceptual consequences of the manipulation of contextual information for visual anticipation.

All in all, in line with the previous findings (Mann et al., 2014; Loffing et al., 2015b; Gredin et al., 2018; Runswick et al., 2019) we found that contextual information influences anticipation performance; particularly decision behavior, but not prediction accuracy to a similar extent. That effect was not accompanied by systematic changes in gaze behavior, at least not in the number of fixations and the duration of the last fixation. Importantly, this does not ultimately mean that there is no effect on a perceptual level and also it would be premature to infer that the effect of action preference on anticipation performance is likely due to biases occurring at later information processing stages. For this purpose, it would be necessary to further investigate how the respective types of information (i.e., kinematic and contextual information) are combined. In this respect, Helm et al. (2020) postulate a Bayesian approach and oppose an “either or strategy” by which they exclude simple heuristics and an equal weighting model as alternative explanations. While the effect of knowledge of an opponent's action preference on anticipation performance appears quite robust (cf. Mann et al., 2014; Loffing et al., 2015b; Gredin et al., 2018; Runswick et al., 2019), identification of its underlying perceptual-cognitive mechanisms remains a challenging task for future experimental research. The latter is suggested relevant for the development of strategies on how to integrate contextual information (e.g., action preferences) into training and match preparation to ensure athletes use this information most effectively (Gray, 2015).

## Data Availability Statement

The datasets generated for this study are available on request to the corresponding author.

## Author Contributions

TL, FL, and JS contributed conception and design of the study. TL organized the database, performed the statistical analysis, and wrote the first draft of the manuscript. FL and JS wrote sections of the manuscript. All authors contributed to manuscript revision, read, and approved the submitted version.

### Conflict of Interest

The authors declare that the research was conducted in the absence of any commercial or financial relationships that could be construed as a potential conflict of interest.

## Footnotes

^1^Three participants were excluded prior to the data analysis. One participant assumed that misinformation was given between block 1 and block 2 and assumed a distribution of 75% longline balls instead. The other two participants were excluded due to technical problems with the mobile eye-tracker during testing.
