# Supplementary material for: On the Influence of Action Preference on Female Players' Gaze Behavior During Defense of Volleyball Attacks
Source: Front Sports Act Living. 2020 Feb 4;2:6. doi: 10.3389/fspor.2020.00006 (PMC7739626; doi:10.3389/fspor.2020.00006)
Supplement: Supplementary file 1 [file Data_Sheet_1.docx]

Supplementary Material

Table S1. Analysis of decision behaviour.

| ***effect*** | ***df*** | ***F*** | ***p*** | ***η_p_²*** |
| --- | --- | --- | --- | --- |
| group | 1, 22 | 6.222 | .021 | .220 |
| block | 1, 22 | 2.332 | .141 | .096 |
| block x group | 1, 22 | 28.843 | < .001 | .567 |
| preference | 1, 22 | 0.916 | .349 | .040 |
| preference x group | 1, 22 | 0.006 | .937 | .000 |
| block x preference | 1, 22 | 0.274 | .606 | .012 |
| block x preference x group | 1, 22 | 2.301 | .144 | .095 |
|  | | | | |

Table S2. Analysis of prediction accuracy.

| ***effect*** | ***df*** | ***F*** | ***p*** | ***η_p_²*** |
| --- | --- | --- | --- | --- |
| group | 1, 22 | 0.909 | .351 | .040 |
| block | 1, 22 | 1.161 | .293 | .050 |
| block x group | 1, 22 | 2.972 | .099 | .199 |
| preference | 1, 22 | 0.229 | .637 | .010 |
| preference x group | 1, 22 | 0.937 | .344 | .041 |
| block x preference | 1, 22 | 0.110 | .743 | .005 |
| block x preference x group | 1, 22 | 2.208 | .152 | .091 |
|  | | | | |

Table S3. Analysis of number of mean fixations.

| ***effect*** | ***df*** | ***F*** | ***p*** | ***η_p_²*** |
| --- | --- | --- | --- | --- |
| group | 1, 22 | 0.349 | .560 | .016 |
| block | 1, 22 | 10.821 | .003 | .330 |
| block x group | 1, 22 | 0.014 | .908 | .001 |
| preference | 1, 22 | 5.426 | .029 | .198 |
| preference x group | 1, 22 | 0.592 | .450 | .026 |
| block x preference | 1, 22 | 0.287 | .597 | .013 |
| block x preference x group | 1, 22 | 1.679 | .208 | .071 |
|  | | | | |

Table S4. Analysis of duration of the last fixation.

| ***effect*** | ***df*** | ***F*** | ***p*** | ***η_p_²*** |
| --- | --- | --- | --- | --- |
| group | 1, 22 | 1.051 | .316 | .046 |
| block | 1, 22 | 0.015 | .905 | .001 |
| block x group | 1, 22 | 0.129 | .723 | .006 |
| preference | 1, 22 | 5.933 | .023 | .212 |
| preference x group | 1, 22 | 2.200 | .152 | .091 |
| block x preference | 1, 22 | 0.056 | .815 | .003 |
| block x preference x group | 1, 22 | 0.230 | .637 | .010 |
|  | | | | |
